# Going back to normal, but how? Re-opening policies and the standards for a new normal

**DOI:** 10.3906/sag-2106-263

**Published:** 2021-12-17

**Authors:** Levent AKIN

**Affiliations:** 1 Department of Public Health, Faculty of Medicine, Hacettepe University, Ankara Turkey

**Keywords:** COVID-19, pandemics, prevention and control, measures, policy

## Abstract

Governments implemented to nonpharmacological methods and various limitation policies such as closing nonessential businesses, schools and limiting group gatherings, promoting social distancing, use of personal protective equipment, advising staying at home. These policies have caused various problems in social and economic life and gradual increase in psychosocial well-being problems. All societies yearningly are waiting for the COVID-19 pandemic to be brought under control and the measures to be lifted in order to return to their previous lives. Indicators are needed to assess the burden of disease in the country while lifting measures to control the COVID-19 pandemic. When using these indicators, it is necessary to consider the own characteristics of the countries. Personal precautions need to be continued for a while until vaccination becomes widespread and effective all over the world.

## 1. Introduction

All over the world the COVID-19 pandemic continues with all its severity, sometimes increasing or decreasing in countries. Currently, there have been approximately 175 million cases and 3.8 million deaths worldwide. As of the beginning of June 2021, there are a total of 13.5 million active cases https://ourworldindata.org/covid-cases / [accessed 06 June 2021].. In order to reduce the burden of disease, governments resorted to nonpharmacological methods and enacted various policies such as closing schools, nonessential businesses and limiting group gatherings, promoting social distancing, use of face-mask, advised stay-at-home [1]. These policies, which were taken since the beginning of 2020, have led to various problems in societies and their gradual increase such as mental health and psychosocial well-being problems, socioeconomic disparities, gender-based violence, discontinuity of health and public health programs, reaching management of chronic diseases other than COVID-19 [2]. Recent research has showed that COVID-19 pandemic affected country economies in various sectors such as consumption, services, finance, industries, and investments. Countries’ economies have become derogate due to public health measures and also caused increasing unemployment in many countries Goodman-Bacon A, Marcus J. Using Difference-in-Differences to Identify Causal Effects of COVID-19 Policies. Berlin, Germany: Deutsches Institut für Wirtschaftsforschung German Institute for Economic Research; 2020. Website ( https://ojs.ub.uni-konstanz.de/srm/article/view/7723, [accessed 6 June 2021].. 

A few countries in their communities are easing public protective measures by speeding up vaccination programs against COVID-19. 

Currently, nonpharmaceutical interventions actually continue, but crucial is balancing between to provide public health safety and lightened social and economic troubles [3]. Nowadays all countries are trying to put forward principles and indicators that will bring social and economic life back to normal, while struggling with the pandemic. Public health protective measures should be assessed periodically and revised according to the needs of community and burden of COVID-19. Assessments should include the level of transmission, the capacity of health system, risky places and forthcoming events, which help to change transmissibility or the burden of the diseases in the community. 

## 2. Effectiveness of interventions

The aim of public health measures is to break the chain of infection; thus, transmission can be prevented from person to person and new cases can be limited further spread of COVID-19, particularly until vaccines and therapeutics are available. The main public health measures are given below:

1. All personal measures recommended inhibiting person-to-person transmission World Health Organization. Critical preparedness, readiness, and response actions for COVID-19. study [online]. Website (https://www.who.int/publications-detail/critical-preparedness-readiness-and-response-actions-for-covid-19, [accessed 6 June 2021].. Personal measures include frequent respiratory etiquette, physical distancing, hand hygiene. As in droplet-transmitted infections, the most important protection in the COVID-19 pandemic is personal protective equipment. The studies showed that odds ratio (OR) for transmission risk of SARS-CoV-2 infection was 0.08 (95% CI 0.02–0.31) and keeping the distance more than 1 m from a COVID-19 case reduced the risk approximately 7 times; OR was found 0.13 (95% CI 0.04–0.46). One of the risks for infection is duration of contact with infected cases. Shortening the contact time with an infected person by less than 15 min reduces OR value by almost half [OR: 0.41 (95% CI 0.18–0.91)]. Hand washing properly is another important protective measure, which reduces the risk of infection by one-fifth, and OR was found to be 0.19 (95% CI 0.08 to 0.46). Adjusted odds ratio of wearing masks correctly or incorrectly (such as not covering both nose and mouth), which were calculated by using logistic regression were 0.23 (95% CI 0.09–0.60) and 0.87 (95% CI 0.41–1.84), respectively [4]. In a review, it has been shown that keeping physical distance as 1 m or more and the use of masks could reduce the risk of COVID19 transmission respectively 10 times and 7–10 times [4]. 

In a modeling study, it was found that the widespread use of masks prevents cases and decreases the proportion of infectors who pass the infection to 5 or more people. Increased mask use may lead to increased mask effectiveness, a reduction in all types of transmission events, and even reduction of super-spread events [5]. 

The widespread use of personal protection measures in the community should be continued until the COVID-19 pandemic is over worldwide in order to prevent the spread of disease. Personal protective equipment should not be associated with reopening nor should it be considered during reopening practices. In terms of the rules of using masks only in open areas, mask usage principals can be arranged according to crowded areas or close contact with people.

Physical and social distancing in public places is to prevent transmission from infected persons to susceptible ones. These are physical distancing, restraining of mass gatherings, and avoiding different crowded places (e.g., public transport, restaurants, cinemas, pubs, places of entertainment), working at home (if appropriate) and staying at home, and closing educational institutions Key planning recommendations for mass gatherings in the context of the current COVID-19 outbreak study [online]. Website (https://www.who.int/publications-detail/key-planning-recommendations-for-mass-gatherings-in-the-context-of-the-current-covid-19-outbreak) [accessed 6 June 2021].. According to the information obtained from the cases with COVID-19 as a result of the research and contact follow-up, being in closed places with more than 10 people is a risk factor for the transmission of the disease. For example, COVID-19 cases were reported more frequently when dining at a restaurant, which can be indoors, outdoors, or patio seating. Within the 2 weeks preceding such activity, an illness onset with an adjusted odds ratio of 2.4 (CI 95% 1.5–3.8) was observed [6]. In a study conducted in the USA, it was calculated that the restaurants that will lead to the highest increase in cases will be opened at full capacity. As a result of the opening of these points, predicted transmission rate might increase up to 2.4 times. In this study, fitness centers, cafes and snack bars, hotels, and motels have been considered the most risky areas [7]. 

2. Limitation of mobility and travel are recommended to prevent spreading of infection from one area to another area. This limitation may also affect airports, bus, and train stations. Restriction of mobility in the community may be a good solution to maintain social distancing and to prevent the spread of the disease. Since social distancing is an effective measure to reduce exposure to the virus, as well as the use of masks and maintaining a certain social distance in public areas. It should be ensured that these cautions are taken to decrease the virus spread, especially within the socially vulnerable groups. People in such groups are likely to be working in various sectors such as the service and industrial sector. Therefore, preventing individuals in such groups from traveling between home and work is not feasible. Either of the following two strategies can be applied. 1-All workplaces are placed in lockdown, 2- People who go to work and their contacts are regularly tested

Another important issue in human movements is traveling by public transports such as trains, buses, which appear to expose their passengers to a higher risk of infection than others (e.g., airplanes) due to longer exposure times and seat-to-seat distance. Epidemiological studies show that case incidence and risk of infection are positively correlated with travel, travel frequency, and longer travel time Vitrano C., COVID-19 and Public Transport, A Review of the International Academic Literature K2 WORKING PAPER 2021:1 study [online]. Website https://www.researchgate.net/publication/348677976_COVID-19_and_Public_Transport_A_Review_of_the_International_Academic_Literature [accessed 4 June 2021]..

Along with the measures taken with the opening of schools, the risk of COVID in schools is also evaluated. The reports from studies suggest that the number of children infected with COVID-19 is less than infected adults. Many studies from various countries show that infection risk of children was low [8]. This situation should be interpreted with caution, since cases may be unobserved because of asymptomatic infections in children, as testing is restricted to symptomatic cases. Current evidence give rise to thought that young children have a weaker role in the dynamics of COVID-19 transmission than confirmed adolescent cases worldwide. Some research suggests that children can be asymptomatic carriers, while other studies have found low transmission rates from children to adults. Older children may transmit at higher rates than younger children Bailey J. Is it Safe to Reopen Schools? An Extensive Review of the Research March 2021 [online]. Website https://www.crpe.org/sites/default/files/final_is_it_safe_to_reopen_schools_an_extensive_review_of_the_research.pdf.

Evaluation of the US CDC surveillance system data is presented in Table 1 Risk for COVID-19 Infection, Hospitalization, and Death By Age Group, Updated Feb. 18, 2021 Center for Diseases Control and Prevention, COVID-19. Website https://www.cdc.gov/coronavirus/2019-ncov/covid-data/investigations-discovery/hospitalization-death-by-age.html [accessed 5 June 2021].. When the 5–17 age group is taken as a reference, it is seen that the risk of infection is lower in the 0–4 age group, but the risk of hospitalization and death is higher. In addition, it is seen that the risk of hospitalization and death is much higher in all age groups compared to this age group, and the infection is higher in the 18–64 age group. Briefly, children have lower risks related to COVID-19 infections, hospitalizations, and deaths compared to adults contracting COVID-19.

**Table 1:** Risk for COVID-19 infection, hospitalization, and death by age group.

	Age groups								
	0-4	5-17	18–29	30–39	40–49	50–64	65–74	74–84	85+
Cases risk	<1x	Reference group	2×	2×	2×	2×	1×	1×	2×
Hospitalization risk	2x	Reference group	6×	10×	15×	25×	40×	65×	95×
Death risk	2x	Reference group	10×	45×	130×	440×	1,300×	3200×	8700×

Available information shows that schools reflect the epidemiologic patterns of infection in their communities. The research on children’s role in transmitting the corona virus is still uncompelling. 

3. Specific protective measures are taken to protect vulnerable groups, which have high risk of severe of disease if they get infection (e.g., older people, persons with comorbidity). Persons or groups with social vulnerabilities (e.g., refugees, displaced populations), groups in closed settings (e.g., long-term living facilities, disabled nursing home, and prison) are at high risk for clustering when any of the residents become infected. Patients who were living in nursing homes or prisons, from ethnic minority backgrounds, admitted to hospital for a long-term health problem in the past 5 years, and living in overcrowded residences were all considered as vulnerable groups, and the age-adjusted COVID-19 death rate increased for such groups by 28%, 19%, 8%, and 11%, respectively [9]. 

4. Health workers and frontline responders were highly exposed to the virus at the workplace. When front-line health-care workers were compared with the general community, risk for a positive COVID-19 test was increased and adjusted hazard ratio was found to be 11.61, (95% CI 10.93–12.33). In other words, the probability of a positive COVID-19 test of health personnel is at least 10 times higher than the general population. Therefore, adequate PPE should be provided to healthcare professionals Nguyen LH, Drew DA., Graham MS., Guo GG. Ma W., Joshi AD., Risk of COVID-19 among front-line health-care workers and the general community: a prospective cohort study [online]. Website https://www.thelancet.com/journals/lanpub/article/PIIS2468-2667(20)30164-X/fulltext [accessed 13 June 2021]..

3. Monitoring and re-opening indicators: The purpose of monitoring is to identify the effects of COVID-19 response activities and to provide strategic information to decision makers on reducing the burden caused by the pandemic. Standardized indicators are needed to be used for monitoring and assessment the situation of COVID-19. Therefore, specific indicators should be constituted to describe the epidemiologic characteristics of COVID-19 in the population, the effects of nonpharmacological and public health response measures and mitigation or reinforcement of certain responses to COVID-19, and early warning indicators for increased COVID-19 transmission [10]. 

There are many indicators while monitoring the COVID-19 pandemic, but easy to apply and effective indicators should be presented to decision makers in determining the level of measures to be taken to introduce, keep or lift the measures in the community. In this regard, WHO and the USA offer different approaches, indicators and methods, although they are for the same purpose. Countries follow their validity and evaluate the effects of the measures of their own countries according to their socioeconomic status, demographic characteristics, the extent of the epidemic, the burden of COVID-19, and spread characteristics in the country.

There are two type of indicators that most commonly used in monitoring the disease burden caused by the COVID-19 pandemic. These are as follows: 

1) The 7-day (or 14-day) incidence rate: The number of daily cases can fluctuate depending on the number of tests performed daily. This can be caused by daily applying to test centers for testing, screenings in clustered-case communities, or even weekend breaks. Therefore, 7-day or 14-day incidences recommended to be used.

2) The percent positivity rate in the tests: Two methods can be used Understanding Percent Positivity, [online]. Website https://publichealthmdc.com/blog/understanding-percent-positivity, [accessed 12 June 2021]..

a) Test over test: The percentage of positivity in all tests in a given period. This method is in use widely. This method counts people who are tested multiple times. For example, if one person is tested 4 times a week, with three tests being positive and one test being negative, test positivity percent is found 75%. If most persons are only getting tested one time, it is acceptable. But when test availability increase and individuals are tested multiple times, this method loose sensitivity to understand the stage of the pandemic. Classifications of “test over test” 7-Days incidence indicators are shown in Table 2. 

**Table 2:** 7-days incidence indicators by WHO and countries.

Source	Incidence level (weekly per 100,000)			
	Low	Moderate	Moderately high	High
WHO WHO (2020), Considerations for implementing and adjusting public health and social measures in the context of COVID-19, Interim guidance, 4 November 2020 [online]. Website https://www.who.int/publications/i/item/considerations-in-adjusting-public-health-and-social-measures-in-the-context-of-covid-19-interim-guidance [accessed 8 June 2021].	<20	20 to <50	50 to <150	≥150
Kentucky (USA)[11]	≤10	>10 to 49.99	≥50 to 100	>100
USA (ADL data systems) COVID-19 Alert-Level System Indicators, Triggers and Thresholds. [online]. Website https://preventepidemics.org/wp-content/uploads/2020/05/Annex-2_Example-of-an-alert-level-system_US_FINAL.pdf [accessed 01 June 2021]. per 1 million population per day	<10	10-19	20-39	≥40
USA (threshold for school opening)*	5 to <20*	20 to <50	50 to ≤ 200	>200
Scotland’s strategic framework Scotland’s Strategic Framework, A levels approach to suppression of COVID 19, 25 October 2020 [online]. Website https://www.gov.scot/binaries/content/documents/govscot/publications/factsheet/2020/10/coronavirus-covid-19-protection-levels-updated-draft-27-october-2020/documents/indicators-paper/indicators-paper/govscot%3Adocument/Indicators%2Bpaper%2B26%2BOct%2B1645.pdf [accessed 15 June 2021].	20 to 75	75 to 159	150 to 300	>300

*Lowest risk of transmission in schools is <5.

b) People over people: The percentage of positivity individuals in the total number of people who tested both positive and negative. This method does not count duplicate tests, but the number of retest does not account. For example, first test was negative, later than person becomes back and test result positive. This case would be added to the numerator as a new positive, but the denominator would not change because those were counted as same person being tested. 

In addition to these, the indicators used are listed as follows:

· Percent change in new cases per 100,000 population during the last 7 days compared with the previous 7 days, 

· Inpatient beds proportional occupancy by patients with COVID-19,

· ICU proportional occupancy by patients with COVID-19,

· Sudden increase in the number of COVID-19 cases in a localized community or geographic area. 

WHO recommended that the response capacity of the existing health system also needs to be evaluated. For this purposes, proportion of occupied hospital beds, outcome of hospitalized cases, and case-specific fatality rate, number of persons tested per 1000 population per week, proportion of cases for which an investigation has been conducted within 24 h of identification should be considered. 

4. How to lift measures and approach to reopen: The current policies are intended to get slower the transmission of the virus by decreasing contact among individuals and encouragement to use personal protective equipment. It was observed that public health measures have had an impact on limiting transmission of COVID-19 and reducing deaths. However, the effects of these policies on infection rates are not measurably clear during the ongoing pandemic. The decision to introduce, continue or ease public health measures should be based on a situational assessment of the transmission level of virus and the capacity of the health system to respond, by considering in the effects. Indicators and thresholds which are in above are recommended to evaluate both the intensity of transmission and the capacity of the health system to respond. Public health measures must be continuously adjusted to the intensity of transmission and capacity of the health system in a country and at provincial levels.

The criteria to be used should be shared with the public, and the community should be informed about how the measures in the community will change according to the status of the criteria. It should be done with mathematical models using existing surveillance information, and a roadmap should be defined on how to change the measures to be taken according to these models. Thus, both individuals in the society and sector representatives can be informed about what kind of situation they may encounter in which period and their needs can be met. 

Countries should modify measures based on the assessment of the transmission status of the disease in the community, the current capacity of the health system, and the results of research on mathematical modeling. The roadmap published by the UK is a good example in this regard Prime Minister sets out roadmap to cautiously ease lockdown restrictions, Press release 22 February 2021. [online]. Website https://www.gov.uk/government/news/prime-minister-sets-out-roadmap-to-cautiously-ease-lockdown-restrictions[accessed 15 March 2021].. This roadmap consisted of four criteria to ease restrictions: infection rates, situation of new variants of concern, the success of vaccination program, and evidence on vaccines effective in reducing hospitalizations and deaths. 

The suggested principles of public health measures to be applied according to the public burden of the disease are given below:

1. If epidemic is in uncontrolled phase and substantial excess morbidity and mortality, reducing transmission in the community will be challenging, and stricter mobility restrictions and related measures may need to be implemented.

2. The incidence is high, and there is a risk that health services will be stuck. Public health measures should be implemented to limit transmission in the community. At this level, nonessential businesses should be closed and remote work encouraged. It may be necessary for all individuals to reduce their social contact and suspend some activities (gathering, indoor activities and services) while basic services are allowed and schools remain open if appropriate. 

3. If the epidemic is controlled through effective public health measures and incidence is moderate level, but still cases or clusters of cases cause disruption to social life and economic, public health measures should continue, and individual measures should be strengthened. In this situation it should be encouraged to avoid closed places, crowded places, and close-contact settings. Daily activities and services, such as educational settings and work-places can remain open with public health measures to limit the risk of spread. Long-term care facilities should continue by ensuring appropriate measures.

4. If the incidence is low or clusters still are seen, it means that the risk of transmission in the community continues. At this level, measures should be implemented to reduce the contact possibilities of individuals in the community such as staying open with educational environments with security measures, encouraging workplaces with teleworking as much as possible, strictly continuing personal precautionary measures, limiting social and other crowded gatherings. As communities reopen, efforts to reduce possible exposures at locations that offer on-site eating and drinking options should be considered to protect customers, employees, and communities [7]. 

5. If transmission was prevented and only sporadic cases reported the last 28 days, the health system capacity is relieved to respond, but there should be no need restrictions on daily activities. At this level, surveillance should continue to detect any new cases and notify as soon as possible. Basic individual prevention measures and behaviors should stand. Isolation and quarantine are undertaken if cases are confirmed and contacts are followed. Travel can be permitted but travelers from higher incidence areas should be paid attention to.

## 3. Conclusion

Besides the illnesses and deaths caused by the pandemic in societies all over the world, psychosocial problems and economic problems are growing as a result of the protective measures applied in the community, and countries are trying to reduce all the effects caused by the epidemic as soon as possible. Although vaccination is a hope in reducing the effects of the epidemic, the inability of many countries to reach the vaccine causes both increasing the problems of the people living in that country and the emergence of new variants that can change the effectiveness of the vaccine.

## Informed consent

This manuscript is an invited review article and does not consist any experimental investigations with humans, and no institutional review board approval is needed.
